# A T1-hypointense intracranial dermoid cyst

**DOI:** 10.31744/einstein_journal/2021AI6347

**Published:** 2021-12-15

**Authors:** Marcos Gil da Veiga, Amets Sagarribay, Carlos Marques Pontinha, Carla Conceição

**Affiliations:** 1 Hospital Dona Estefânia Centro Hospitalar Universitário de Lisboa Central Lisboa Portugal Hospital Dona Estefânia, Centro Hospitalar Universitário de Lisboa Central, Lisboa, Portugal.; 2 Centro Hospitalar Universitário de Lisboa Central Lisboa Portugal Centro Hospitalar Universitário de Lisboa Central, Lisboa, Portugal.

**Figure 1:**
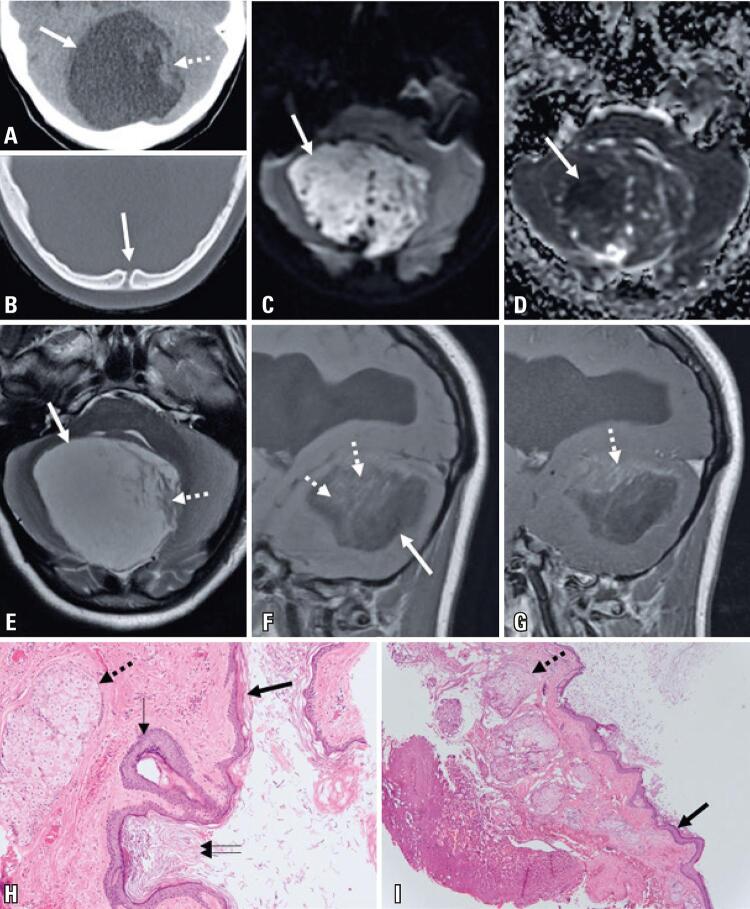
Computed tomography and magnetic resonance illustrating the main imaging features of the dermoid cyst. Microscopy photographs of the lesion. (A) Soft tissue window computed tomography demonstrating the presence of a posterior fossa cystic lesion, mostly hypodense (≃10 Hounsfield Units) (block arrow), with a few slightly denser elements in the left region (≃29 Hounsfield Units) (dotted arrow); (B) Bone window computed tomography demonstrating the presence of a sinus tract, representing a clue to the presence of a possible inclusion cyst; (C and D) Large area of reduced water diffusion, demonstrated by hyperintensity in diffusion-weighted imaging and hypointensity in apparent diffusion coefficient map; (E) Axial T2-weighted image, demonstrating a predominantly T2-hyperintense posterior fossa lesion (block arrow), with a small and irregular superior-posterior T2-isointense region (dotted arrow); (F) Sagittal T1-weighted image, demonstrating a predominantly T1-hypointense posterior fossa lesion (block arrow) with a few superior isointense elements (dotted arrows); (G) Sagittal T1-weighted image, demonstrating the presence of a few linear hyperintense components after gadolinium administration; (H and I) Histology images demonstrating a cystic lesion delineated by a keratinizing squamous epithelium (block arrow) with granular layer (thin arrow), sebaceous glands (dotted arrows) and wet keratin (double arrow), making the diagnosis of dermoid cyst

A 15-year old women presented with a 6-month history of progressive right-hand tremor with functional impairment, aggravated by a 1-month history of episodic confusion. The patient underwent a computed tomography scan, which revealed a posterior fossa expansive lesion with cerebrospinal fluid-like density and a midline bone discontinuity ( Figures 1A and 1B ; soft tissue window not shown). A magnetic resonance imaging (MRI) scan ( Figures 1C to 1G ) was performed after hospital admission, revealing a posterior fossa expansile lesion with predominant T2 hyperintensity, T1 hypointensity, linear and irregular areas of faint enhancement after gadolinium injection, and a large area with reduced water diffusion. Supratentorial images revealed signs of chronic hydrocephalus. The patient underwent surgery revealing a whitish extra-axial capsulated lesion containing dermal appendages, and histology confirmed the diagnosis of a dermoid cyst ( Figures 1H and 1I ).

Intracranial dermoid cysts are rare lesions, representing less than 0.5% of primary intracranial tumors.^( 1 )^ They are congenital ectodermal inclusion cysts and tend to occur in the midline.^( 1 )^ In rarer occasions in which they develop in the posterior fossa, they tend to locate in the vermis or within the fourth ventricle.^( 1 )^ Dermoid cysts may be asymptomatic for a long time and present with a long history of vague symptoms, most commonly headache.^( 2 , 3 )^ Depending on location, they may be associated with focal neurologic *deficits* , seizures, and also recurrent aseptic meningitis.^( 3 )^

Dermoid cysts are classically described as T1-hyperintense lesions, and some authors claim that “all” lesions present that MRI signal.^( 1 )^ These intracranial cysts are also described as lesions without gadolinium enhancement and with apparent diffusion coefficient values similar to brain parenchyma.^( 1 - 4 )^ Some reports describe uncommon imaging features, such as T1-hypointensity, reduced water diffusion or gadolinium-enhancing regions.^( 2 - 4 )^ A dermal sinus may present as a clinical/imaging clue to the correct diagnosis.^( 2 )^
