# Supplementary material for: A T1-hypointense intracranial dermoid cyst
Source: Einstein (Sao Paulo). 2021 Dec 15;19:eAI6347. doi: 10.31744/einstein_journal/2021AI6347 (PMC8687640; doi:10.31744/einstein_journal/2021AI6347)
Supplement: Translation

# Translation: Cisto dermoide intracraniano hipointenso em T1

**Authors:** Marcos Gil da Veiga, Amets Sagarribay, Carlos Marques Pontinha, Carla Conceição

**Affiliations:** 1 Portugal Hospital Dona Estefânia, Centro Hospitalar Universitário de Lisboa Central, Lisboa, Portugal.; 2 Portugal Centro Hospitalar Universitário de Lisboa Central, Lisboa, Portugal.

Paciente do sexo feminino, 15 anos, com histórico de 6 meses de tremor progressivo na mão direta com disfunção funcional, agravada por histórico de 1 mês de ataque episódico. A paciente foi submetida a rastreamento por tomografia computadorizada, que revelou lesão expandida da fossa posterior com densidade similar a de fluido raquimedular e descontinuidade óssea na linha mediana ( Figuras 1A e 1B ; janela de tecido mole não demonstrada). O exame de imagem por ressonância magnética (RM) ( Figuras 1C a 1G ) foi realizado após internação hospitalar, revelando lesão expandida na fossa posterior, com predominância de hiperintensidade T2, hipointensidade T1 e áreas lineares com redução de difusão de água. As imagens supratentoriais reveleram sinais de hidrocéfalo crônico. A paciente foi submetida à cirurgia, na qual se observou lesão capsulada extra-axial com substância branca contendo apêndices cutâneos, sendo confirmado o diagnóstico de cisto dermoide na histologia ( Figuras 1H e 1I ).

**Figura 1:** Tomografia computadorizada e ressonância magnética ilustrando as principais características da imagem de cisto dermoide. Imagens microscópicas da lesão. (A) Tomografia computadorizada em janela de tecido mole indicando a presença de lesão cística da fossa posterior, na maioria hipodensa (≃10 unidades Hounsfield) (seta), com pequenos elementos densos na região esquerda (≃29 unidades Hounsfield) (seta tracejada); (B) Tomografia computadorizada em janela óssea demostrando a presença de fístula, representando possibilidade de presença de cisto de inclusão; (C e D) Área extensa de redução da difusão de água, demostrada por hiperintensidade de imagem ponderada em difusão e hipointensidade em coeficiente de mapa de difusão aparente; (E) Imagem axial ponderada em T2, demostrando T2 hiperintenso posterior na lesão da fossa predominante (seta); (F) Imagem sagital ponderada em T1, demostrando um T1 hipointenso posterior da lesão da fossa posterior predominante (seta) com pequenos elementos isointensos superiores (setas tracejadas); (G) Imagem sagital ponderada em T1, mostrando presença de componentes hiperintensos lineares após administração de gadolínio; (H e I) Imagens histológicas apresentando lesão cística delineada por epitélio escamoso queratinizado (seta) com camadas granulosas (seta fina), glândulas sebáceas (seta tracejada) e queratina molhada (seta dupla), tornando possível o diagnóstico de cisto dermoide

Cistos intracranianos são lesões raras e representam menos do que 0,5% dos tumores intracranianos primários.^( 1 )^Esses são cistos ectodérmicos de inclusão congênita e tendem a ocorrer na linha mediana.^( 1 )^Em ocasiões raras em que esses são desenvolvidos na fossa posterior, eles tendem a se localizar no *vermis* ou no quarto ventrículo.^( 1 )^Os cistos dermoides podem ser assintomáticos a longo prazo e apresentar histórico longo de sintomas vagos, sendo muito comum a dor de cabeça.^( 2 , 3 )^Dependendo do local, tais sintomas podem estar associados a *deficits* focais neurológicos, convulsões e, também, meningite asséptica recorrente.^( 3 )^

Os cistos dermoides são classicamente descritos como lesões T1 hiperintensa, e alguns autores afirmam que “todas” as lesões são apresentadas em sinais de RM.^( 1 )^Esses cistos intracranianos também estão descritos como lesões sem melhora de gadolínio e com difusão aparente de valores de coeficientes similares aos do parênquima cerebral.^( 1 - 4 )^Alguns relatos descrevem características de imagem incomuns, como hipointesidade T1, redução de difusão de agua ou regiões melhora de gadolínio.^( 2 - 4 )^A fístula dérmica pode se apresentar como indicação clínica/imagem para o diagnóstico correto.^( 2 )^
